# A locus at 19q13.31 significantly reduces the *ApoE* ε4 risk for Alzheimer’s Disease in African Ancestry

**DOI:** 10.1371/journal.pgen.1009977

**Published:** 2022-07-05

**Authors:** Farid Rajabli, Gary W. Beecham, Hugh C. Hendrie, Olusegun Baiyewu, Adesola Ogunniyi, Sujuan Gao, Nicholas A. Kushch, Marina Lipkin-Vasquez, Kara L. Hamilton-Nelson, Juan I. Young, Derek M. Dykxhoorn, Karen Nuytemans, Brian W. Kunkle, Liyong Wang, Fulai Jin, Xiaoxiao Liu, Briseida E. Feliciano-Astacio, Alzheimer’s Disease Sequencing Project, Alzheimer’s Disease Genetic Consortium, Gerard D. Schellenberg, Clifton L. Dalgard, Anthony J. Griswold, Goldie S. Byrd, Christiane Reitz, Michael L. Cuccaro, Jonathan L. Haines, Margaret A. Pericak-Vance, Jeffery M. Vance

**Affiliations:** 1 John P. Hussman Institute for Human Genomics, Miller School of Medicine, University of Miami, Miami, Florida, United States of America; 2 Dr. John T. Macdonald Foundation Department of Human Genetics, University of Miami, Miller School of Medicine, Miami, Florida, United States of America; 3 Department of Psychiatry, Indiana University School of Medicine, Indianapolis, Indiana, United States of America; 4 College of Medicine, University of Ibadan, Ibadan, Nigeria; 5 Department of Biostatistics and Health Data Science, Indiana University School of Medicine, Indianapolis, Indiana, United States of America; 6 Department of Genetics and Genome Sciences, Case Western Reserve University, Cleveland, Ohio, United States of America; 7 Universidad Central del Caribe, Bayamón, Puerto Rico, United States of America; 8 Penn Neurodegeneration Genomics Center, Department of Pathology and Laboratory Medicine, University of Pennsylvania Perelman School of Medicine, Philadelphia, Pennsylvania, United States of America; 9 Department of Anatomy, Physiology & Genetics, Uniformed Services University of the Health Sciences, Bethesda, Maryland, United States of America; 10 Maya Angelou Center for Health Equity, Wake Forest School of Medicine, Winston-Salem, North Carolina, United States of America; 11 Gertrude H. Sergievsky Center, Taub Institute for Research on the Aging Brain, Departments of Neurology, Psychiatry, and Epidemiology, College of Physicians and Surgeons, Columbia University, New York, New York State, United States of America; 12 Department of Population & Quantitative Health Sciences, Cleveland Institute for Computational Biology, Case Western Reserve University School of Medicine, Cleveland, Ohio, United States of America; HudsonAlpha Institute for Biotechnology, UNITED STATES

## Abstract

African descent populations have a lower Alzheimer disease risk from *ApoE* ε4 compared to other populations. Ancestry analysis showed that the difference in risk between African and European populations lies in the ancestral genomic background surrounding the *ApoE* locus (local ancestry). Identifying the mechanism(s) of this protection could lead to greater insight into the etiology of Alzheimer disease and more personalized therapeutic intervention. Our objective is to follow up the local ancestry finding and identify the genetic variants that drive this risk difference and result in a lower risk for developing Alzheimer disease in African ancestry populations. We performed association analyses using a logistic regression model with the *ApoE* ε4 allele as an interaction term and adjusted for genome-wide ancestry, age, and sex. Discovery analysis included imputed SNP data of 1,850 Alzheimer disease and 4,331 cognitively intact African American individuals. We performed replication analyses on 63 whole genome sequenced Alzheimer disease and 648 cognitively intact Ibadan individuals. Additionally, we reproduced results using whole-genome sequencing of 273 Alzheimer disease and 275 cognitively intact admixed Puerto Rican individuals. A further comparison was done with SNP imputation from an additional 8,463 Alzheimer disease and 11,365 cognitively intact non-Hispanic White individuals. We identified a significant interaction between the *ApoE* ε4 allele and the SNP rs10423769_A allele, (β = -0.54,SE = 0.12,p-value = 7.50x10^-6^) in the discovery data set, and replicated this finding in Ibadan (β = -1.32,SE = 0.52,p-value = 1.15x10^-2^) and Puerto Rican (β = -1.27,SE = 0.64,p-value = 4.91x10^-2^) individuals. The non-Hispanic Whites analyses showed an interaction trending in the “protective” direction but failing to pass a 0.05 significance threshold (β = -1.51,SE = 0.84,p-value = 7.26x10^-2^). The presence of the rs10423769_A allele reduces the odds ratio for Alzheimer disease risk from 7.2 for *ApoE* ε4/ε4 carriers lacking the A allele to 2.1 for *ApoE* ε4/ε4 carriers with at least one A allele. This locus is located approximately 2 mB upstream of the *ApoE* locus, in a large cluster of pregnancy specific beta-1 glycoproteins on chromosome 19 and lies within a long noncoding RNA, ENSG00000282943.

This study identified a new African-ancestry specific locus that reduces the risk effect of *ApoE ε4* for developing Alzheimer disease. The mechanism of the interaction with *ApoEε4* is not known but suggests a novel mechanism for reducing the risk for *ε4* carriers opening the possibility for potential ancestry-specific therapeutic intervention.

## Author summary

Strong associations between *ApoE* ε4 and Alzheimer disease risk have been confirmed worldwide, but there is variability in the effect size across populations. African-descent populations have a lower risk from *ApoE* ε4 compared to other populations. Studies in admixed populations have shown that the African ancestral background surrounding the *ApoE* gene reduces the ε4 risk allele effect. Our objective in this study was to identify areas of the genome that interact with *ApoE* ε4 in African ancestry and result in a lower risk for developing Alzheimer disease. In this study we identify a protective locus for the *ApoE* ε4 allele that lowers the risk for African carriers of the *ApoE* ε4 allele to get Alzheimer disease from an odds ratio of 7.2 to 2.1. This protective haplotype has a frequency of 12% in the African ancestry, but only 0.003 in Europeans. This has been replicated in three independent African ancestry datasets and is trending in a much larger European dataset.

## Introduction

The apolipoprotein E (*ApoE*) gene (19q13.32) is the strongest genetic risk factor for late-onset Alzheimer disease (AD) and is associated with an earlier age-of-onset [1,2]. Compared to the common ε3 allele, the *ApoE* ε4 allele increases AD risk, while the ε2 allele decreases AD risk (e.g. provides a protective effect) relative to the other two alleles [1–4].

**Fig 1:**
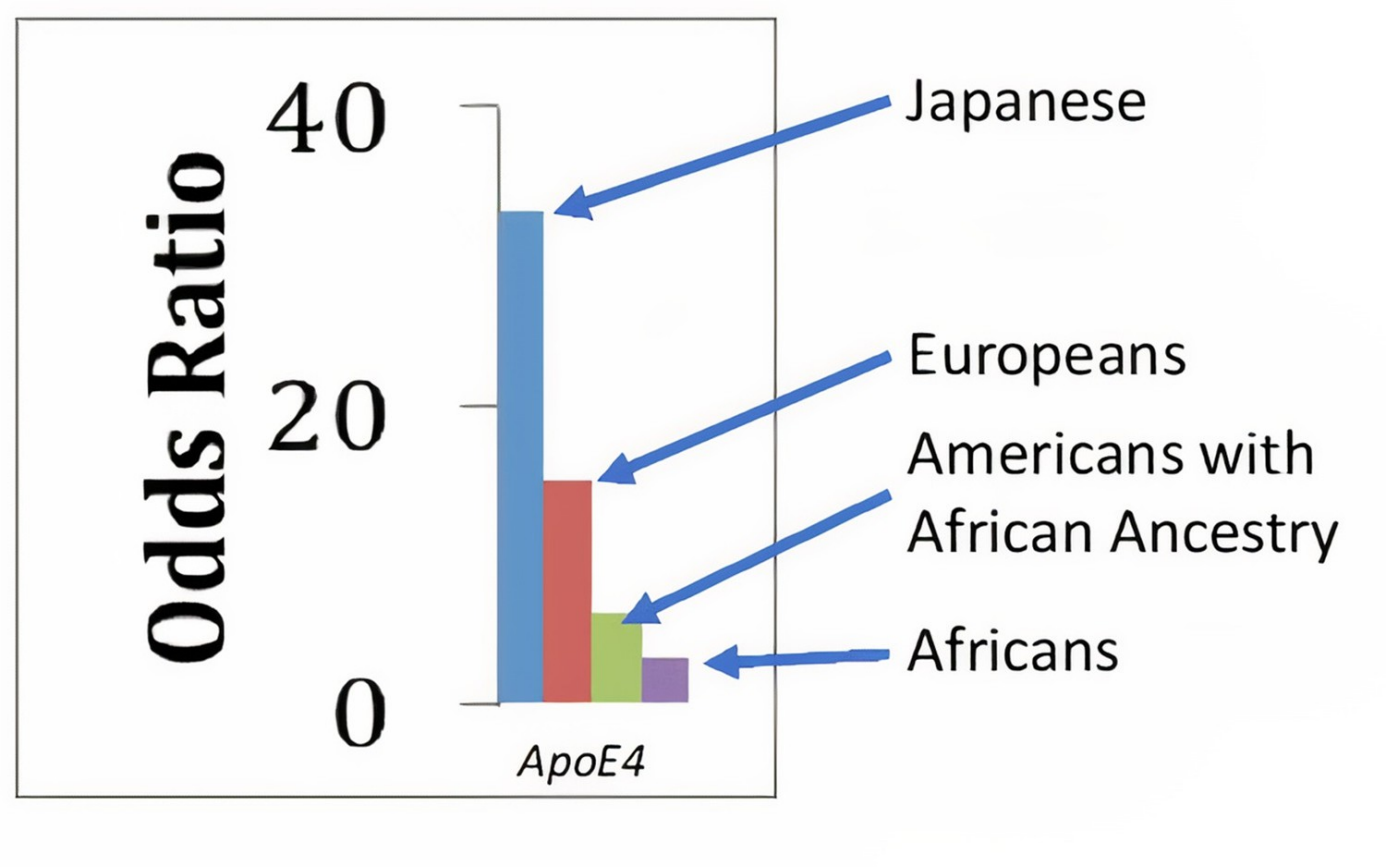
Odds ratios for developing Alzheimer disease according to *ApoE* ε4/ε4 genotypes carriers relative to the ε3/ε3 carriers across the multiple ancestries.

Identifying protective variants against the development of AD has been a key goal of different research groups, including the AD Sequencing Project [5]. The identification of these natural protections may provide insights into disease mechanisms driving AD development as well as potential therapeutic avenues for AD treatment. Indeed, the *ApoE* ε4 allele has a heterogeneous AD risk effect across diverse ancestral populations [3] (Fig 1). The strongest risk effect from *ApoE* ε4 for AD is in East-Asian populations, with the lowest risk from *ApoE* ε4 in African (AF)-Ancestry populations (such as Ibadan individuals from Nigeria and African Americans (AA)) [3,6–10]. This finding suggested the presence of protective genetic loci that modify AD risk associated with the *ApoE* ε4 allele contributing to this difference in population risk. Using admixed populations with the substantial proportion of AF ancestral genetic background (AA, Puerto Rico (PR) and the Dominican Republic), two independent studies [11,12] demonstrated that the difference in risk between AF and European (EU) populations lies in the ancestral genomic background surrounding the *ApoE* locus (local ancestry, or LA). Specifically, when the *ApoE* ε4 allele lies on an AF-originated haplotype the AD risk is significantly lower than if it lies on EU-originating haplotypes. Simply put, an individual who has inherited their *ApoE* ε4 allele from an AF ancestor has the lower *ApoE* ε4-associated AD risk observed in AF populations, while an individual who has inherited their *ApoE* ε4 allele from an EU ancestor has the AD risk observed in EU.

Our objective is to follow up the local ancestry finding and identify the genetic variants that lower the risk for *ApoE* ε4 in African ancestry. We have assessed the *ApoE* ε4 haplotypes of both EU and AF local ancestry using several genomic approaches [13,14]. We report here results of a genetic interaction study that identified an AF-specific haplotype that is associated with a substantially reduced risk for AD in African *ApoE ε4* carriers. This locus lies in the pregnancy specific beta-1 glycoproteins (*PSG*) gene cluster on chromosome 19, approximately two megabases (mB) upstream of the *ApoE* locus.

## Results

**Fig 2:**
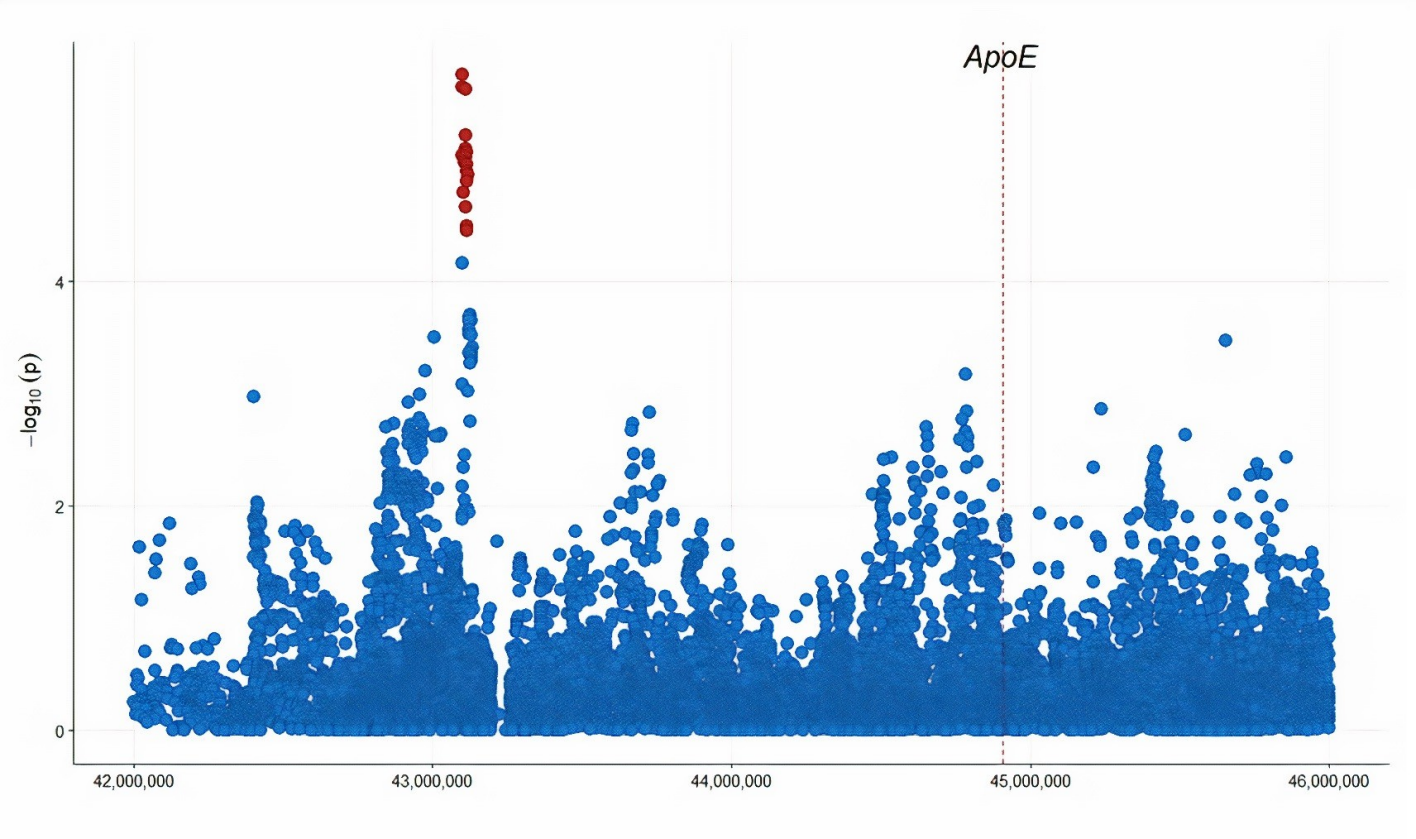
The regional plot of logistic regression analysis across the *ApoE* region for the interaction term. Genomic coordinates are displayed along the X-axis. Negative logarithm of the association p-value for the interaction term is displayed on the Y-axis, with the red color representing markers that have FDR adjusted p-value < 0.05.

We identified a locus (rs10423769-allele A) that lies within a large cluster of *PSG* genes on chromosome 19 approximately 2 mB from the *ApoE* gene. This locus has a significant interaction with the *ApoE* ε4 allele (*β* = −0.54, *SE* = 0.12, *p*−*value* = 7.5×10^−6^) in the AA samples, meeting an FDR correction threshold for multiple testing (*p*−*value* = 0.014). Individuals carrying the minor allele “A” at rs10423769 showed a reduction in AD risk due to *ApoE* ε4. Fig 2 illustrates the logistic regression model results across the *ApoE* region for the interaction term (QQ plot illustrated in S1 Fig). In the replication phase, we performed the epistatic interaction model in an independent cohort of Ibadan individuals from Nigeria. Additionally, we used two diverse datasets to reproduce the effect: a cohort of admixed Hispanic individuals of PR ancestry from the mainland United States and Puerto Rico, and a large collection of non-Hispanic Whites (NHW), primarily from the United States. Results showed a significant interaction between *ApoE* ε4 and rs10423769 in the Ibadan and PR datasets (Ibadan: *β* = −1.32, *SE* = 0.52, *p*−*value* = 1.15×10^−2^; PR: *β* = −1.27, *SE* = 0.64, *p*−*value* = 4.91×10^−2^). The NHW analyses showed an interaction trending in the “protective” direction but failing to pass a 0.05 significance threshold (*β* = −1.51, *SE* = 0.84, *p*−*value* = 7.26×10^−2^) (Table 1). The main effect of the rs10423769 marker was not by itself significantly associated with AD (p-value = 0.46) in the reduced logistic regression model (no interaction term).

**Table 1:** Effects of the rs10423769 genotype and its *ApoE* ε4 allele interaction terms in the logistic regression model across studies.

	*β*	*SE*	*p*−*value*
African American	-0.54	0.12	7.5×10^−6^
Ibadan	-1.32	0.52	1.2×10^−2^
Puerto Rican	-1.27	0.64	4.9×10^−2^
non-Hispanic White	-1.51	0.84	7.3×10^−2^

**Fig 3:**
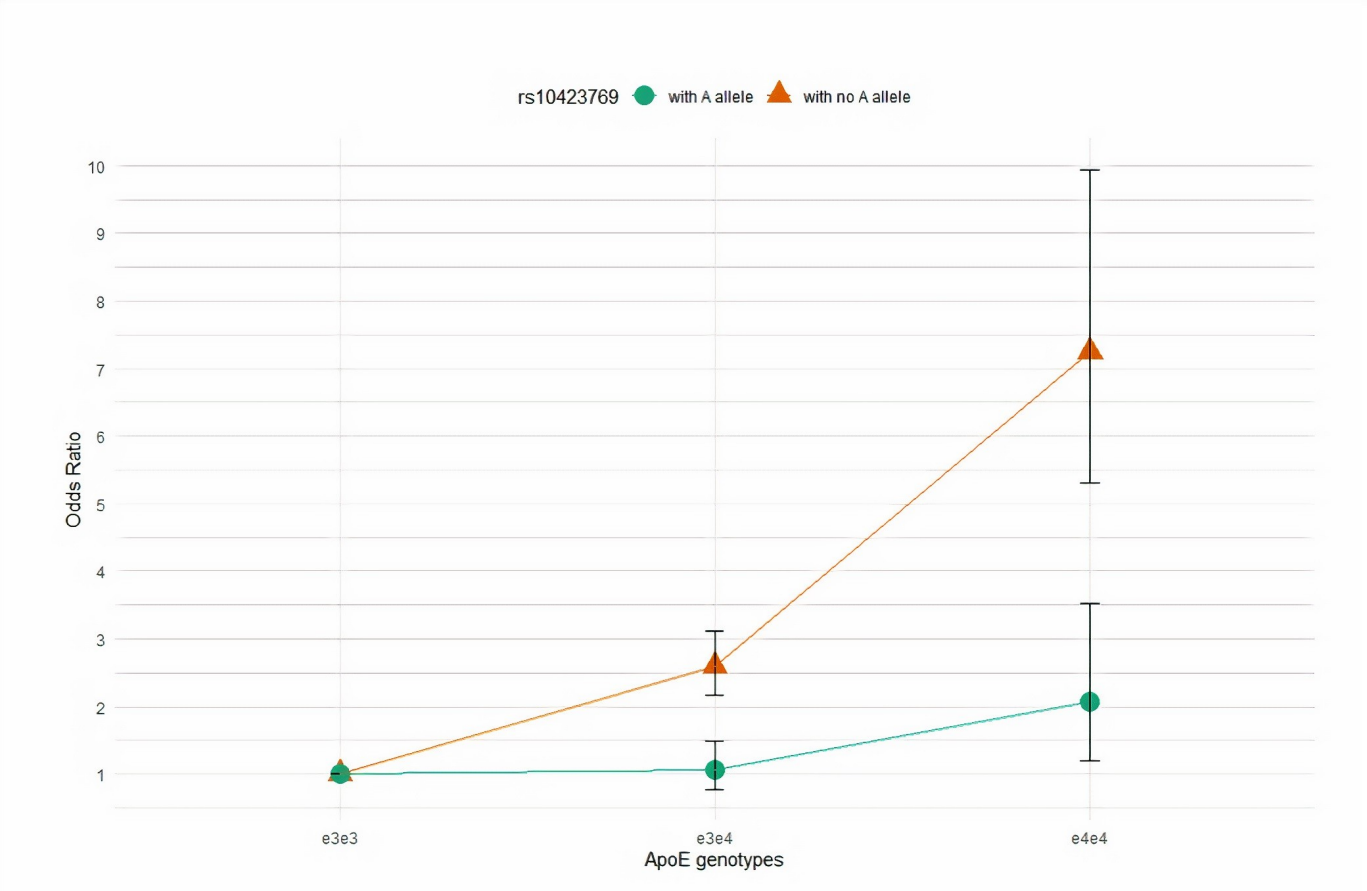
The plot illustrates odds ratios for ε3/ε4 and ε4/ε4 genotypes relative to ε3/ε3 individuals in the combined African American and Ibadan individuals stratified by the rs10423769_A allele. Subgroup odds ratios (95% CIs) are denoted by green color (circle) for rs10423769_A allele carriers and brown color (triangular) for no rs10423769_A allele carriers.

Next, we investigated the modifier effect of the *ApoE*ε4 risk allele for AD in individuals with the homozygous AF local ancestry within two subgroups stratified by the rs10423769_A allele. In one subgroup, we included individuals homozygous for the “G” allele and in the other we included individuals with at least one alternative “A” allele. To assess the AD risk effect of *ApoE* genotypes ε3/ε4 and ε4/ε4 relative to the ε3/ε3 genotype, we restricted the sample set to those that were not ε2 allele carriers. Only four PR individuals were identified with the rs10423769_A allele and homozygous AF local ancestry, so we did not include individuals from PR for further analysis. We performed logistic regression analysis within AA, Ibadan populations, and in combined AA and Ibadan individuals separately. Then, we tested the risk effect size differences of ε3/ε4 and ε4/ε4 genotypes using two-sample z-test. We found that in the subgroup of rs10423769_A allele carriers the effect sizes of ε3/ε4 and ε4/ε4 genotypes were significantly lower than in the non-carriers (AA: ε3/ε4: *p*−*value* = 1.43×10^−5^; ε4/ε4: *p*−*value* = 8.79×10^−4^; Ibadan: ε3/ε4: *p*−*value* = 0.033; ε4/ε4 were absent in cases of rs10423769_A allele carriers; AA and Ibadan individuals combined: ε3/ε4: *p*−*value* = 5.70×10^−6^; ε4/ε4: *p*−*value* = 7.11×10^−5^). Odds Ratios for developing AD according to *ApoE* genotypes stratified by the rs10423769_A allele in AA and Ibadan populations are shown in Table 2. Fig 3 illustrates the AD risk effect of ε3/ε4 and ε4/ε4 genotypes relative to the ε3/ε3 in combined AA and Ibadan individuals across the strata of rs10423769 genotypes.

**Table 2:** Odds Ratios for developing Alzheimer disease according to ApoE genotypes ε3/ε4 and ε4/ε4 relative to the ε3/ε3 stratified by the rs10423769_A allele and studies.

		African American			Ibadan			African American + Ibadan		
		N	OR (95% CI)	p-value	N	OR (95% CI)	p-value	N	OR (95% CI)	p-value
rs10423769_A carriers	ε3/ε3	300	1(Referent)		92	1(Referent)		392	1(Referent)	
	ε3/ε4	250	1.06(0.74–1.51)	0.757	60	0.49(0.10–1.91)	0.337	310	1.07(0.86–1.61)	0.681
	ε4/ε4	55	2.41(1.34–4.35)	0.003	12	…*	…	67	2.06(1.21–3.52)	0.008
rs10423769_A non-carriers	ε3/ε3	1139	1(Referent)		244	1(Referent)		1383	1(Referent)	
	ε3/ε4	934	2.61(2.15–3.17)	3.59x10^-22^	157	2.83(1.40–5.87)	0.004	1091	2.59(2.16–3.12)	2.37x10^-24^
	ε4/ε4	187	7.58(5.43–10.69)	8.97x10^-32^	20	5.02(1.26–17.0)	0.013	207	7.24(5.31–9.94)	3.12x10^-35^

*ε4/ε4 genotype data were absent in cases

**Fig 4:**
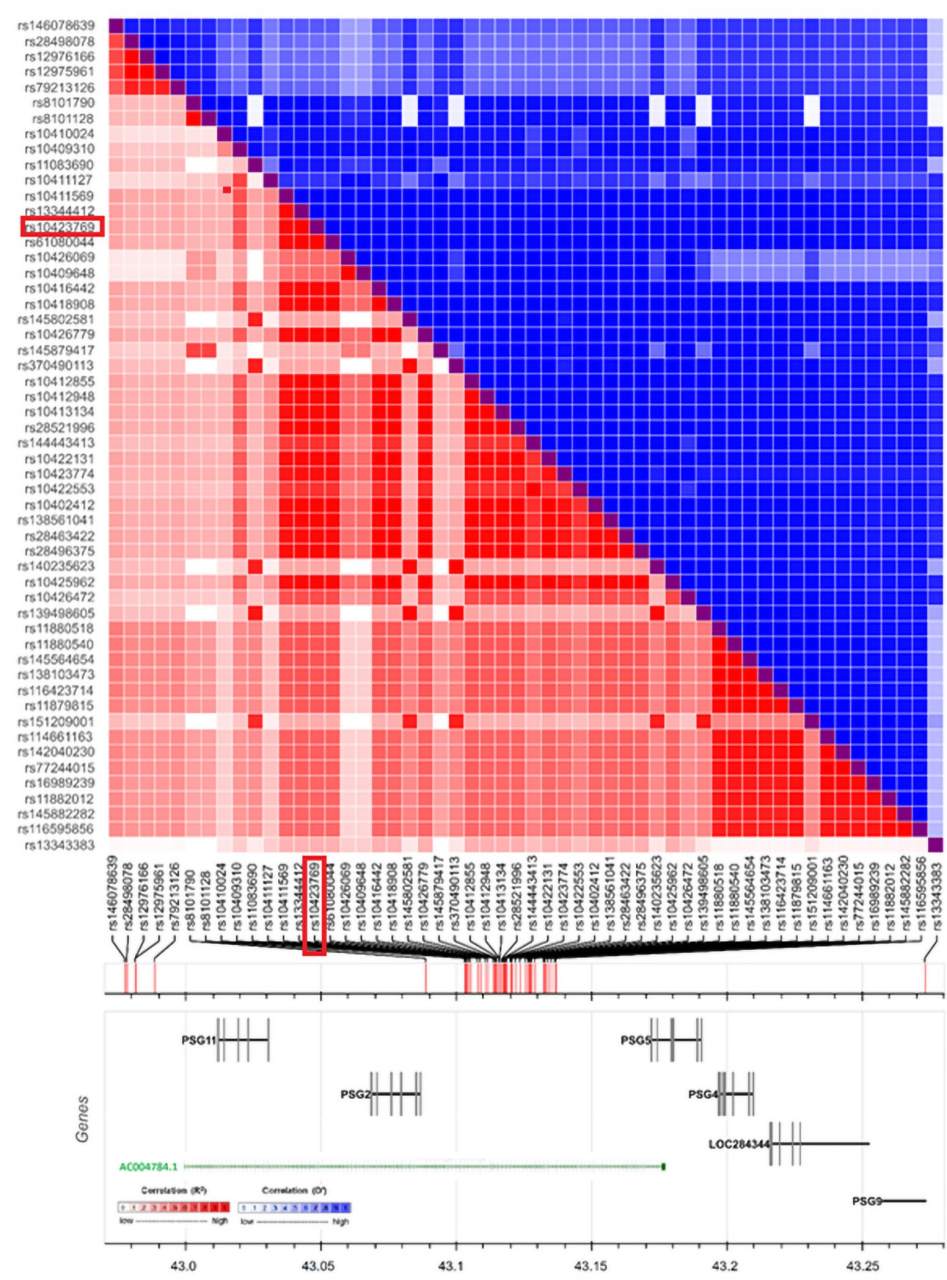
The plot illustrates the Linkage Disequilibrium (LD) heatmap surrounding the rs10423769 variant. The LD matrix was constructed based on r^2^(red color squares) and D′ (blue color squares) measurement for all pairs of variants. “Genes” panel illustrates the genes that are overlapping with the LD pattern.

The AF haplotype associated with the “A” allele for rs10423769 is shown in Fig 4. The haplotype lies 18kb upstream of the *PSG2* gene. This haplotype lies within the long noncoding gene ENSG00000282943 (also identified as *AC004784*.*1* and *CTC-490G23*.*6*), primarily expressed in the cerebellum and fibroblasts based on data from GTEx [15].

Splicing quantitative trait loci (sQTL) analysis using the GTEx database shows that rs10423769 is a significant sQTL for the *TMEM145* gene in the cerebellum with the “A” allele having a 1.6-fold increase in splicing levels between chr19:42320437 and chr19:42320653 relative to the “G” allele (*p*−*value* = 2.7×10^−6^).

**Fig 5:**
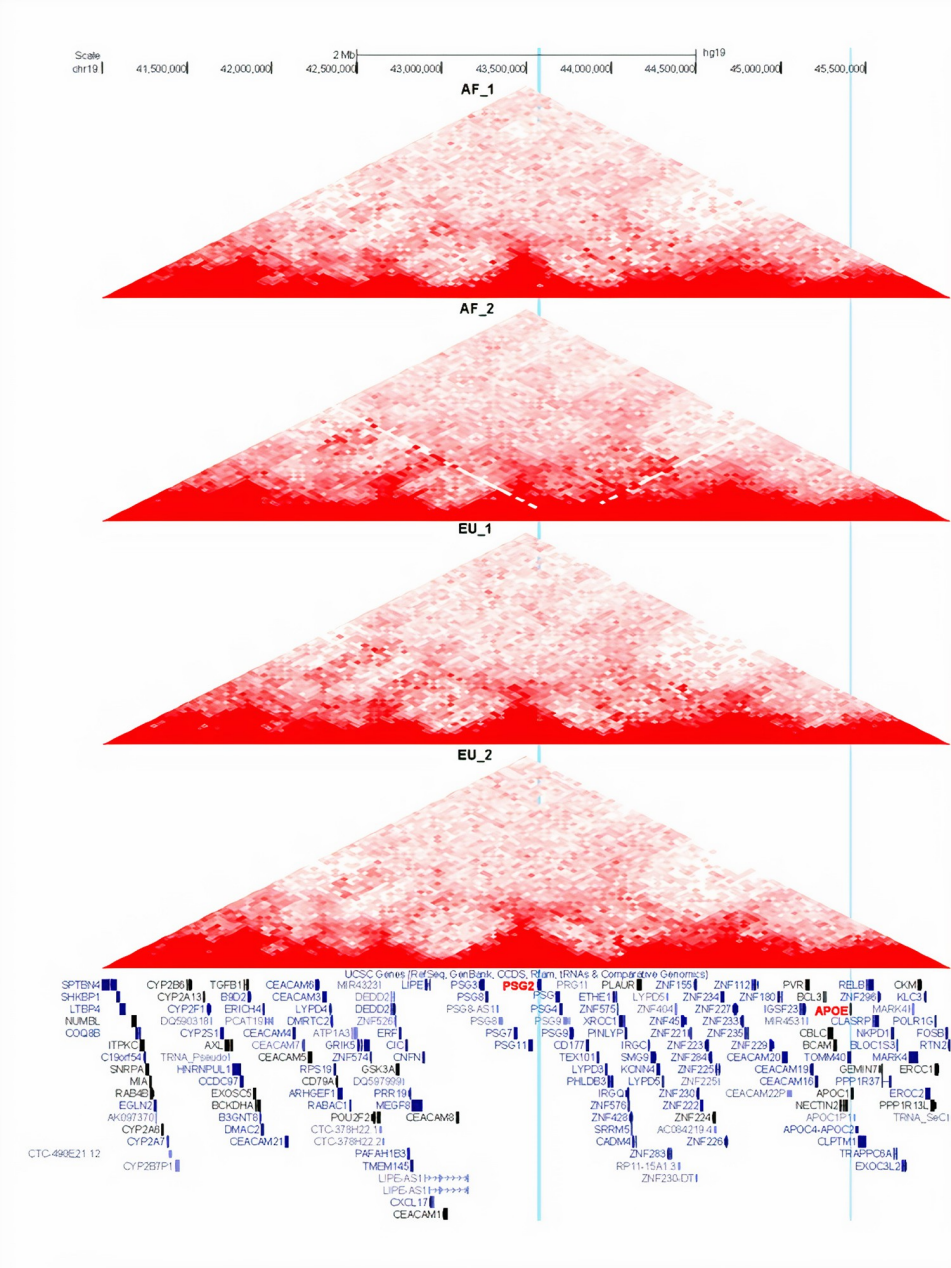
Chromatin Loops in chromosome 19 region centering on *PSG2* and *ApoE* genes are displayed in four tracks, each representing one astrocyte line. Induced pluripotent stem cells (iPSCs) with African (20–1611720 and LWHiC, top two tracks) or non-Hispanic White local ancestry (20–1616981 and 201–806023, bottom two tracks) were differentiated into astrocytes. Chromatin Hi-C library was constructed with 4-cutter enzyme. Blue vertical bar highlights rs10423769; yellow vertical bar highlights *ApoE*.

We used Hi-C analysis to investigate if the locus represented by rs10423769 directly interacts with *ApoE* gene locus via cis 3D chromatin looping. As *ApoE* is primarily expressed in astrocytes and microglia, we performed Hi-C analysis in iPSC-derived astrocytes from *ApoE* ε4 homozygotes who were homozygous for either African or European local ancestry surrounding *ApoE* ε4. As shown in Fig 5 *PSG2* and *ApoE* reside in separate topologically associated domains (TADs) and no cis enhancer-promoter loop was detected between the two loci in either local ancestry. However, cerebellum data were not available.

## Discussion

This study identified a new AF ancestry-specific haplotype that reduces the AD risk effect of *ApoE ε4* homozygotes in AF ancestry by approximately 75%. Previous studies have shown that the African local ancestral background of the *ApoE* gene reduces the AD risk due to the *ε4* allele, with individuals inheriting the *ApoE ε4* allele from African ancestors having a lower risk of AD than individuals inheriting the *ApoE ε4* allele from European ancestry [11,12]. Our results corroborate with these findings and identify a novel African locus (19q13.31**)** that explains a portion of the lower risk due to *ApoE ε4* in African local ancestry individuals. A recent single-nuclei RNA study showed that European local ancestry carriers had significantly higher *ApoE* expression than African local ancestry carriers, suggesting ancestral-specific regulation of *ApoE* gene expression [13] could be contributing to this risk difference as well. These two findings suggest a polygenic modulation of the *ε4* allele risk effect among populations.

Local ancestry blocks across the genome have a wide distribution in size. In our study’s largest dataset of AA individuals (3000) the mean of local ancestry block sizes across the genome was ~36Mb (S2 Fig). The local ancestry region in the Rajabli et al. study [11] has an ad-hoc definition of 1 mB on either side of the *ApoE* gene to functionally include the topological associated domains surrounding *ApoE*. The current study expands the Rajabli et al study to a wider genetic region that includes +/-3 mB around the *ApoE* gene, but still correlates with the previously identified effect of the local ancestry associated with differences in risk for AD between *ApoE* ε4 carriers.

Since we detected a statistical interaction between *PSG2* and *ApoE* in individuals with African ancestry, we asked if the two loci have cis interaction with each other that could explain the statistical finding. Towards this end, we constructed a chromatin 3D interaction map in iPSC-derived astrocytes with African local ancestry and European local ancestry surrounding *ApoE* using Hi-C. No evidence of cis interaction was observed in this cell type, which along with microglia, is the major cell that expresses *ApoE*. As most enhancers interact within 1 mB [16], it is unlikely that other cell types in African ancestry would contain an interaction at the distance separating rs10423769 and *ApoE* is (~2 mB) but can’t be completely ruled out.

Indeed, the distance between the two interacting loci suggests that other mechanisms than enhancer-promoter maybe involved in this protective effect. The protective haplotype overlies the long noncoding RNA ENSG00000282943, but little is known about its specific function. Interestingly, rs10423769 is reported to be a sQTL for *TMEM145*, which has not been implicated in AD, although it has been reported to be upregulated in anterior cingulate cortex in Dementia with Lewy body patients [17]. Interestingly, while both loci have low expression throughout the brain, their highest brain expression is in the cerebellum, particularly for *TMEM145*. The cerebellum has had observed changes in AD, especially in early onset forms of the disease. But it has received little investigation in the pathophysiology of AD, as historically the cerebellum has been studied for its role in the regulation of motor activity. However, recent studies have shown a role for the cerebellum in cognition, including roles in working memory and executive and visuospatial functions [18–20].

The protective haplotype also lies within a large cluster of *PSG* genes, a family of glycoproteins that are primarily synthesized in the syncytiotrophoblast of the human placenta. Several case-control studies have shown that low levels of *PSG2* are associated with pre-eclampsia [21], which, in turn, has been suggested to be associated with an increased risk of dementia later in life [22]. Indeed, a recent study demonstrated that inducible pluripotent stem cell (iPSC) derived neurons made from the blood of autopsy confirmed AD patients, had abnormal tau deposition which matched their autopsy findings [23]. As these are very young neurons, it raises the possibility that the processes that evidentially lead to AD could begin very early in life. Thus, although they appear temporally distant from the onset of clinical AD in late life, their involvement cannot be entirely ruled out.

This finding is the first AF specific protective effect in AD and highlights the importance of diversity and the inclusion of all populations into research. It hopefully will encourage additional studies focused on diverse populations, where allelic frequency differences can discover information that is hidden when studying only a single population. With most clinical trials in AD ending in failure, the identification of natural protective interactions is of key importance in moving therapeutic efforts in AD forward for all ancestries. Finally, as *ApoE* ε4 is the major genetic risk factor, this work further supports growing efforts to explore it as a major therapeutic target for AD.

## Materials and methods

### Ethics statement

Written consent was obtained from all participants and study protocols were approved by the University of Miami Institutional Review Board (IRB), the Indiana University IRB and the IRB of the University of Pennsylvania.

### Study samples

Our study consisted of a discovery phase using TOPMed imputed genotype data from AA individuals in nine datasets [24] and a replication phase with whole genome sequencing (WGS) data from Ibadan individuals from Nigeria [25]. Additionally, we used PR individuals [26], as well as TOPMed imputed genotype data from non-Hispanic/Latino White (NHW) individuals in 31 datasets [27] to reproduce the findings in diverse datasets. The characteristics of each post-QC datasets are shown in Table 3. The detailed description of the datasets is provided in S1 Appendix and elsewhere [24–28].

**Table 3:** Characteristics of African American, Ibadan, Puerto Rican, and non-Hispanic White data sets.

Characteristic	African American	Ibadan	Puerto Rican	non-Hispanic White
Individuals, No.				
Cases	1850	63	273	8463
Controls	4331	648	275	11365
Women, No. (%)				
Cases	1290 (69.7)	52 (82.5)	181 (66.3)	4710 (55.7)
Controls	3120 (72.0)	401 (61.9)	213 (77.5)	6678 (58.8)
Age, mean (SD)				
Cases^a^	78.6(8.1)	83.5(5.2)	76.5(8.3)	75.9(6.9)
Controls^b^	75.9(8.4)	81.1(4.5)	75.4(6.5)	77.5(7.3)
*ApoE* ε4 frequencies, No. (%)				
-/- ^c^	3553 (57.5)	432 (60.8)	383 (69.9)	12218 (61.6)
-/ ε4	2280 (36.9)	246 (34.6)	144 (26.3)	6627 (33.4)
ε4/ ε4	348 (5.6)	33 (4.6)	23 (3.8)	983 (5.0)

^a^ Age on onset

^b^ Age at last evaluation

^c^ Containing genotypes ε2/ ε2, ε2/ ε3 and ε3/ ε3

#### Genotyping and sequencing

Genome-wide single-nucleotide polymorphism (SNP) genotyping was processed on multiple different platforms and *ApoE* genotyping was performed in the individual datasets as summarized in the S1 Table, S1 Methods and elsewhere [24–28].

Samples from Ibadan and PR had WGS performed at the Uniformed Services University of the Health Sciences (USUHS) using standard protocols as previously described [29]. Illumina’s HiSeq Alignment Software (HAS) was used to analyze the data including alignment to the GRCh38 reference genome with the Issac aligner [30] and variant calling with Strelka [31]. Illumina’s gvcfgenotyper was used to merge the resulting gvcfs into a cohort level vcf. Variant calls for the positions used in the replication phase had sequencing depth of coverage greater than 15X and an alternate allele fraction between 35% and 65% for heterozygotes and >95% for homozygotes.

Standard quality control (QC) for genotype and individual-level data were performed for each dataset using software PLINK v.2 [32]. Variants with the call score less than 98%, or not in Hardy-Weinberg equilibrium (HWE) (p<1.e-6) were eliminated. Individuals with genotyping call rates less than 97% were removed. Individuals whose reported sex differed from the genotype-inferred sex by analysis of the X-chromosome SNPs were excluded. The relatedness among the individuals within and across the case/control datasets was identified by the estimated proportion of alleles (π) shared identical by descent (IBD), and one individual from relatives (π>0.4) was included for the analysis. Population substructure was evaluated in each cohort separately using EIGENSTRAT software [33]. Population substructure in each data sets were compared with those in the 1000 Genome reference panel YRI (Yoruba from Nigeria) and CEU (Utah Residents with Northern and Western European ancestry) populations. Outliers with respect to CEU population (overlapping within the cluster of CEU) were removed from the datasets [34]. S3 Fig illustrates principal component analysis for each African American dataset.

#### Genotype imputation

We imputed AA and NHW genotype array datasets individually using the TOPMed R2 version panel (build of GRCh38) and TOPMed Imputation server [35]. The TOPMed R2 reference panel has 97,256 samples and provides information on 308,107,085 genetic variants [36]. Most of the samples in the TOPMed panel are non-EU and around 25% of the samples are from AF-descent populations. We kept the high-quality common variants (R^2^ > 0.8) with a minor allele frequency (MAF) > 0.05 in the existing AA datasets.

#### Assessment of genetic ancestry

We calculated global ancestry (principal components (PC)) within each array dataset using the EIGENSTRAT approach using EIGENSOFT software with no reference population [33]. To estimate the local ancestry in AA and PR datasets, we first combined each of the array datasets with the Human Genome Diversity Project (HGDP) reference panel separately using PLINK v2 software [32,37]. We used 98 AF, 109 EU, and 108 Amerindian individuals from HGDP reference populations. Then, we phased combined datasets using the SHAPEIT tool ver. 2 with default settings and the 1000 Genomes Phase 3 reference panel [34,38]. Finally, we inferred the local ancestries using the discriminative modeling approach implemented in RFMix with the PopPhased option and a minimum node size of 5 [39].

### Statistical analysis

#### Identifying protective loci

To identify protective loci that modify the *ApoE* ε4 risk effect, we performed an interaction analysis in our discovery AA datasets, focusing on the broad genetic region that includes +/-3 mB around the *ApoE* gene. Imputed data were force-called to the most likely genotype (with 0.90 threshold for the probability) and then assessed using a logistic regression approach. Our primary model for the logistic regression included AD status as the outcome (dependent) variable. Independent variables included force-called genotype and *ApoE* ε4 main effects along with an interaction term between genotype and *ApoE* ε4. Additional covariates included age, sex, and genome-wide ancestry (PC1:3) (Eq 1). We coded both the variants and *ApoE* ε4 allele under a dosage model (0,1,2) and performed interaction analysis in each imputed AA datasets separately. Then, we meta-analyzed all terms across AA datasets by applying fixed-effect meta-analysis (assuming similar effect sizes) from METASOFT software [40]. We used the Benjamini and Hochberg approach to control for the false discovery rate (FDR) [41]. Subsequently, replication analyses were performed using WGS datasets on Ibadan and PR individuals and meta-analysis in 31 imputed genotype datasets from NHWs.


AD∼Age+Sex+PC1:3+ε4+Variant+ε4×Variant 1

#### Assessing modifiers

To assess the influence of putative modifiers on *ApoE* ε4 risk effect, we compared the AD risk effect of *ApoE* ε3/ε4 and ε4/ε4 genotypes relative to the ε3/ε3 genotype in those that were carriers of the modifier alleles to those that were not carriers. First, we restricted the sample set to those that were homozygous for the AF genetic ancestry around the *ApoE* locus and were not *ApoE* ε2 allele carriers. We then stratified by carrier status at loci identified as FDR significant. Next, we performed logistic regression with *ApoE* genotypes (ε3/ε3 (reference), ε3/ε4, ε4/ε4), age, sex, principal components (PC1-3), and batch as covariates within each group (carriers and non-carriers of the modifier allele) for each study and across studies. Finally, we tested the difference in effect sizes of the *ApoE* genotypes between carriers and non-carriers of the putative modifiers using two-sample z-test. Statistical analyses were performed using the “GLM2” package available in R computing environment [42].

#### Hi-C analysis

Hi-C analysis was performed on astrocytes derived from induced pluripotent stem cells (iPSCs) derived from AD patients who were *ApoE* ε4/ε4 and had AF ancestry. Cells were differentiated and cultured using the StemDiff Astrocyte Differentiation and Maturation kits (StemCell Technologies) according to the manufacturer’s protocol. *In situ* Hi-C libraries were prepared using the protocol adapted from Rao et al. [43]. For each library, 450–550 million paired-end reads at 150 bp length were obtained. Sequencing data were processed using BWA to map each read end separately to GRCh38 reference genome [44]. Duplicate and non-uniquely mapped reads were removed. For each library, over 270 million of non-redundant, uniquely mapped, paired reads were used for further analysis. Contact matrices were generated at base pair delimited resolutions of 50 kb [45].

## Supporting information

S1 Appendix Description of datasets. (DOCX)

S1 Methods Diagnosis of AD and age of onset and Genotyping. (DOCX)

S1 Table Genotyping platforms used in individual datasets. (DOCX)

S2 Table Effects of the rs10423769 genotype and *ApoE* ε4 allele interaction term in the logistic regression model for individual African American datasets. (DOCX)

S1 Fig QQ plot and the lambda value of the interaction term in the primary logistic regression model. (DOCX)

S2 Fig Distribution of admixture block sizes in ~3,000 African American individuals. Admixture block sizes are displayed along the x-axis in log_10_ scale.
(DOCX)

S3 Fig Principal component analysis for each African American dataset combined with 1000 Genome CEU and YRI reference populations. (DOCX)
