# Supplementary material for: A locus at 19q13.31 significantly reduces the ApoE ε4 risk for Alzheimer’s Disease in African Ancestry
Source: PLoS Genet. 2022 Jul 5;18(7):e1009977. doi: 10.1371/journal.pgen.1009977 (PMC9286282; doi:10.1371/journal.pgen.1009977)
Supplement: S1 Appendix — (DOCX) [file pgen.1009977.s001.docx]

**eAppendix.** Description of datasets

This study included individuals from the Adult Changes in Thought (ACT) Study [1], the National Institute on Aging (NIA) Alzheimer‘s Disease Centers (ADCs)[2], the University of Miami/Vanderbilt University (UM/VU)[3,4], the Mount Sinai School of Medicine (MSSM) Brain Bank [5], the Washington Heights Inwood Columbia Aging Project (WHICAP)[6], The African American Alzheimer's Disease Genetics (AAG) Study[7], NIA-LOAD/NCRAD[8], the Mayo Clinic[9], the Rush University Alzheimer’s disease Center (ROS/MAP, Minority Aging Research Study/Clinical Minority Core (MARS/CORE))[10-13], the Chicago Health and Aging Project (CHAP)[14,15], the Indianapolis Ibadan Dementia Study (Indianapolis)[16], the University of Pittsburgh (UP)[17], Washington University (WU)[18-21], and Replication and Extension of ADSP Discoveries in African-Americans[22]. Detailed description of each cohort explained elsewhere[23].

All study participants provided written informed consent; for those with significant cognitive impairment, the legal guardian provided informed consent. All study protocols were approved by the corresponding institutional review boards[23].

**Reference:**

1. Kukull WA, Higdon R, Bowen JD, et al. Dementia and Alzheimer disease incidence: a prospective cohort study. Arch Neurol. 2002;59(11):1737-1746. doi:noc20207 [pii]
2. Beekly DL, Ramos EM, Lee WW, et al. The National Alzheimer’s Coordinating Center (NACC) database: the Uniform Data Set. Alzheimer Dis Assoc Disord. 2007;21(3):249- 258. doi:10.1097/WAD.0b013e318142774e00002093-200707000-00009 [pii]
3. Beecham GW, Martin ER, Li YJ, et al. Genome-wide association study implicates a chromosome 12 risk locus for late-onset Alzheimer disease. Am J Hum Genet. 2009;84(1):35-43. doi:10.1016/j.ajhg.2008.12.008
4. Edwards TL, Scott WK, Almonte C, et al. Genome-wide association study confirms SNPs in SNCA and the MAPT region as common risk factors for Parkinson disease. Ann Hum Genet. 2010;74(2):97-109. doi:AHG560 [pii]10.1111/j.1469-1809.2009.00560.x
5. Haroutunian V, Perl DP, Purohit DP, et al. Regional distribution of neuritic plaques in the nondemented elderly and subjects with very mild Alzheimer disease. Arch Neurol. 1998;55(9):1185-1191.
6. Tang MX, Stern Y, Marder K, et al. The APOE-epsilon4 allele and the risk of Alzheimer disease among African Americans, whites, and Hispanics. *JAMA*. 1998;279(10):751-755.
7. Meier IB, Manly JJ, Provenzano FA, et al. White matter predictors of cognitive functioning in older adults. J Int Neuropsychol Soc. 2012;18(3):414-427. doi:10.1017/S1355617712000227
8. Lee JH, Cheng R, Graff-Radford N, Foroud T, Mayeux R, National Institute on Aging Late-Onset Alzheimer’s Disease Family Study G. Analyses of the National Institute on Aging Late-Onset Alzheimer’s Disease Family Study: implication of additional loci. Arch Neurol. 2008;65(11):1518-1526. doi:10.1001/archneur.65.11.1518
9. Carrasquillo MM, Zou F, Pankratz VS, et al. Genetic variation in PCDH11X is associated with susceptibility to late-onset Alzheimer’s disease. Nat Genet. 2009;41(2):192-198. doi:10.1038/ng.305
10. Barnes LL, Shah RC, Aggarwal NT, Bennett DA, Schneider JA. The Minority Aging Research Study: ongoing efforts to obtain brain donation in African Americans without dementia. Curr Alzheimer Res. 2012;9(6):734-745. <https://www.ncbi.nlm.nih.gov/pubmed/22471868>.
11. Bennett DA, Schneider JA, Bienias JL, Evans DA, Wilson RS. Mild cognitive impairment is related to Alzheimer disease pathology and cerebral infarctions. Neurology. 2005;64(5):834-841. doi:64/5/834 [pii]10.1212/01.WNL.0000152982.47274.9E
12. Bennett DA, Schneider JA, Buchman AS, Mendes de Leon C, Bienias JL, Wilson RS. The Rush Memory and Aging Project: study design and baseline characteristics of the study cohort. Neuroepidemiology. 2005;25(4):163-175. doi:NED2005025004163 [pii]10.1159/000087446
13. Bennett DA, Wilson RS, Schneider JA, et al. Natural history of mild cognitive impairment in older persons. Neurology. 2002;59(2):198-205. http://www.ncbi.nlm.nih.gov/entrez/query.fcgi?cmd=Retrieve&db=PubMed&dopt=Citation &list_uids=12136057.
14. Bienias JL, Beckett LA, Bennett DA, Wilson RS, Evans DA. Design of the Chicago Health and Aging Project (CHAP). J Alzheimers Dis. 2003;5(5):349-355. http://www.ncbi.nlm.nih.gov/pubmed/14646025. Accessed April 20, 2016.
15. Evans DA, Bennett D a, Wilson RS, et al. Incidence of Alzheimer disease in a biracial urban community: relation to apolipoprotein E allele status. Arch Neurol. 2003;60(2):185- 189. doi:noc10242 [pii]
16. Murrell JR, Price B, Lane KA, et al. Association of apolipoprotein E genotype and Alzheimer disease in African Americans. Arch Neurol. 2006;63(3):431-434. doi:10.1001/archneur.63.3.431
17. Kamboh MI, Minster RL, Demirci FY, et al. Association of CLU and PICALM variants with Alzheimer’s disease. Neurobiol Aging. 2012;33(3):518-521. doi:10.1016/j.neurobiolaging.2010.04.015
18. Berg L, McKeel Jr. DW, Miller JP, et al. Clinicopathologic studies in cognitively healthy aging and Alzheimer’s disease: relation of histologic markers to dementia severity, age, sex, and apolipoprotein E genotype. Arch Neurol. 1998;55(3):326-335.
19. Morris JC, Roe CM, Xiong C, et al. APOE predicts amyloid-beta but not tau Alzheimer pathology in cognitively normal aging. Ann Neurol. 2010;67(1):122-131. doi:10.1002/ana.21843
20. Morris JC, Weintraub S, Chui HC, et al. The Uniform Data Set (UDS): clinical and cognitive variables and descriptive data from Alzheimer Disease Centers. Alzheimer Dis Assoc Disord. 2006;20(4):210-216.
21. Storandt M, Grant EA, Miller JP, Morris JC. Longitudinal course and neuropathologic outcomes in original vs revised MCI and in pre-MCI. Neurology. 2006;67(3):467-473. doi:67/3/467
22. Kunkle BW, Carney RM, Kohli MA, Naj AC, Hamilton-Nelson KL, Whitehead PL, Wang L, Lang R, Cuccaro ML, Vance JM, Byrd GS, Beecham GW, Gilbert JR, Martin ER, Haines JL, Pericak-Vance MA. Targeted sequencing of ABCA7 identifies splicing, stop-gain and intronic risk variants for Alzheimer disease. Neurosci Lett. 2017 May 10;649:124-129. doi: 10.1016/j.neulet.2017.04.014. Epub 2017 Apr 8. PMID: 28400126.
23. Kunkle B., et al. Novel Alzheimer Disease Risk Loci and Pathways in African American Individuals Using the African Genome Resources Panel. 2021. *JAMA Neurol.* 78(1):102-113.
