# Supplementary material for: A locus at 19q13.31 significantly reduces the ApoE ε4 risk for Alzheimer’s Disease in African Ancestry
Source: PLoS Genet. 2022 Jul 5;18(7):e1009977. doi: 10.1371/journal.pgen.1009977 (PMC9286282; doi:10.1371/journal.pgen.1009977)
Supplement: S1 Methods — (DOCX) [file pgen.1009977.s002.docx]

**eMethods.**

**Diagnosis of AD and age of onset**

Affected individuals met National Institute of Neurological and Communicative Disorders and Stroke-Alzheimer's Disease and Related Disorders Association criteria for AD[1-3]. The assessment of all unaffected individuals was similar to cases. In most datasets, information on age at onset for cases and age at exam or death for controls was available. However, for some datasets, surrogate age information was available including age at diagnosis (CHAP, MARS/CORE), age at ascertainment (Indiana University), or age at death (subset of autopsy-confirmed samples in the UM/VU dataset). We excluded controls with the age younger than 60 years*.*

**Genotyping**

Genome-wide genotyping arrays are summarized in the Supplementary table 1.

***ApoE* genotyping**

To determine *ApoE* genotypes: for the ADCs, ACT, NIA-LOAD/NCRAD, UM/VU, CHAP, Columbia University, REAAADI, and Mayo Clinic cohorts haplotypes derived from single-nucleotide polymorphisms (SNPs) rs7412 and rs429358; for the GenerAAtions cohort, Roche Diagnostics LightCycler 480 instrument (Roche Diagnostics) and LightMix Kit ApoE C112R R158 (TIB MOLBIOL) were used; for the UP, and Indianapolis cohorts, pyrosequencing or analysis of restriction fragment length polymorphisms were used; for the Religious Orders Study/Rush Memory and Aging Project (ROS/MAP) and MARS/CORE, high-throughput sequencing of codons 112 and 158 in *ApoE* by Agencourt Bioscience Corporation was used; for the WU samples Taqman-based assay from Applied Biosystems was used [4].

**Reference:**

1. McKhann G, Drachman D, Folstein M, Katzman R. Clinical diagnosis of Alzheimer's disease: report of the NINCDS-ADRDA Work Group under the auspices of Department of Health and Human Services Task Force on Alzheimer's Disease. Neurology.1984;34(7): 939–44.
2. McKhann GM, Knopman DS, Chertkow H, et al. The diagnosis of dementia due to Alzheimer's disease: recommendations from the National Institute on Aging-Alzheimer's Association workgroups on diagnostic guidelines for Alzheimer's disease. *Alzheimers Dement.* 2011; 3, 263-269.
3. Albert MS, Dekosky ST, Dickson D, et al. The diagnosis of mild cognitive impairment due to Alzheimer’s disease: Recommendations from the National Institute on Aging-Alzheimer’s Association workgroups on diagnostic guidelines for Alzheimer’s disease. *Alzheimers Dement.*2011;7:270–9.
4. Kunkle B., et al. Novel Alzheimer Disease Risk Loci and Pathways in African American Individuals Using the African Genome Resources Panel. 2021. *JAMA Neurol.* 78(1):102-113.
