# Supplementary material for: A locus at 19q13.31 significantly reduces the ApoE ε4 risk for Alzheimer’s Disease in African Ancestry
Source: PLoS Genet. 2022 Jul 5;18(7):e1009977. doi: 10.1371/journal.pgen.1009977 (PMC9286282; doi:10.1371/journal.pgen.1009977)
Supplement: S1 Table — (DOCX) [file pgen.1009977.s003.docx]

**Supporting Information Table 1**. Genotyping platforms used in individual datasets

| **Dataset** | **Platform** |
| --- | --- |
| ADC1/2 | Human660W-Quad_v1 |
| ADC3 | HumanOmniExpress-12v1 |
| ADC8 | HumanOmniExpressExome-8 v1.2 |
| ACT | Illumina 660k |
| CHAP | Illumina 1 M |
| Indianapolis | Illumina 1 M |
| NIA-LOAD/NCRAD | Illumina 610k and 370k |
| ADGC [2013]* | Illumina 1Mduo (v3) |
| ADGC [2018a]ǂ | Illumina 1Mduo (v3) |
| ADGC [2018b]**  REAAADI | Illumina Global Screening Array  Illumina Global Screening Array v2 |

* includes samples from the AAG Study, ADCs, CHAP, Mayo Clinic, MSSM, NIA-LOAD/NCRAD,

ROS/MAP/MARS/CORE, UM/VU, UP, WHICAP and WU

ǂ includes samples from Mayo Clinic, Kamboh, WU, WHICAP, CHAP, AAG Study

** includes samples from UM/VU, North Carolina A&T, ROS/MAP/MARS/CORE
