# Supplementary material for: A locus at 19q13.31 significantly reduces the ApoE ε4 risk for Alzheimer’s Disease in African Ancestry
Source: PLoS Genet. 2022 Jul 5;18(7):e1009977. doi: 10.1371/journal.pgen.1009977 (PMC9286282; doi:10.1371/journal.pgen.1009977)
Supplement: S2 Table — (DOCX) [file pgen.1009977.s004.docx]

**Supporting Information Table 2:** Effects of the rs10423769 genotype and ApoE ε4 allele interaction term in the logistic regression model for individual African American datasets

| **Dataset** | **p-value** | **beta** | **stdErr** | **Sample Size** |
| --- | --- | --- | --- | --- |
| ACT | 0.032 | -3.019 | 1.410 | 87 |
| ADC1_2 | 0.723 | 0.367 | 1.034 | 114 |
| ADC3 | 0.738 | -0.247 | 0.738 | 212 |
| ADC8 | 0.339 | -0.325 | 0.339 | 684 |
| ADGC_CHOP | 0.0007 | -0.597 | 0.177 | 2199 |
| CHAP | 0.935 | 0.038 | 0.466 | 495 |
| INDIANAPOLIS | 0.008 | -0.884 | 0.335 | 1112 |
| JHU | 0.292 | -0.460 | 0.437 | 396 |
| REAAADI | 0.155 | -0.567 | 0.399 | 882 |
