# Supplementary material for: A locus at 19q13.31 significantly reduces the ApoE ε4 risk for Alzheimer’s Disease in African Ancestry
Source: PLoS Genet. 2022 Jul 5;18(7):e1009977. doi: 10.1371/journal.pgen.1009977 (PMC9286282; doi:10.1371/journal.pgen.1009977)
Supplement: S1 Fig — (DOCX) [file pgen.1009977.s005.docx]

**
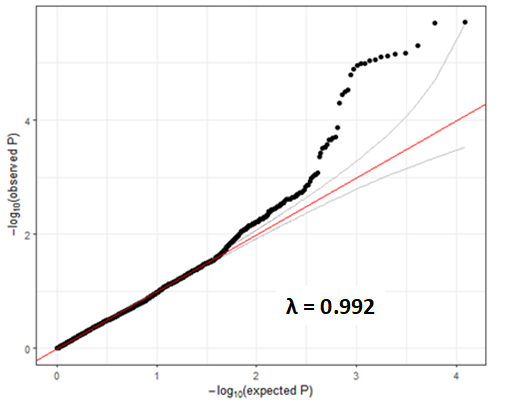
**

**Supporting Information Figure 1:** QQ plot and the lambda value of the interaction term in the primary logistic regression model.
