# Supplementary material for: A locus at 19q13.31 significantly reduces the ApoE ε4 risk for Alzheimer’s Disease in African Ancestry
Source: PLoS Genet. 2022 Jul 5;18(7):e1009977. doi: 10.1371/journal.pgen.1009977 (PMC9286282; doi:10.1371/journal.pgen.1009977)
Supplement: S2 Fig — Admixture block sizes are displayed along the x-axis in log10 scale. (DOCX) [file pgen.1009977.s006.docx]

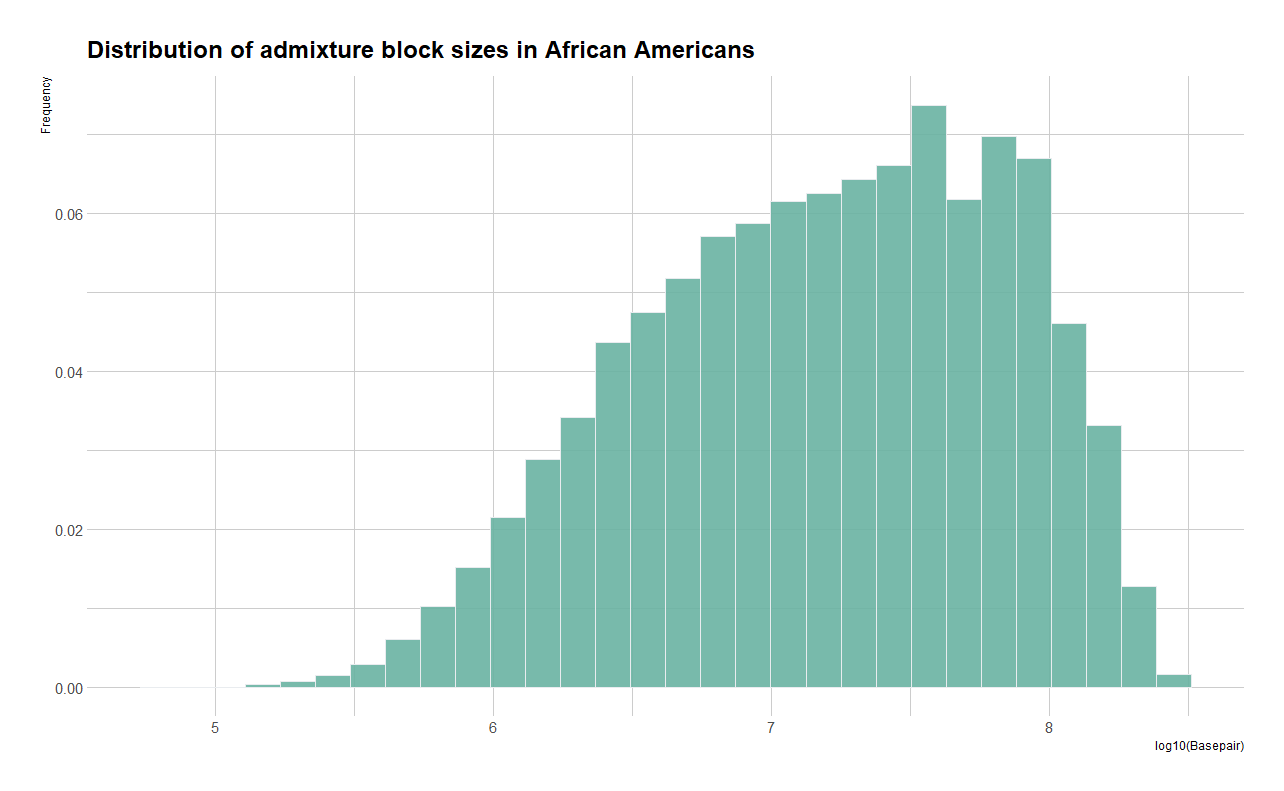


**Supporting Information Figure 2:** Distribution of admixture block sizes in ~3,000 African American individuals. Admixture block sizes are displayed along the x-axis in log_10_  scale.
